# Supplementary material for: Establishment of a UPLC-PDA/ESI-Q-TOF/MS-Based Approach for the Simultaneous Analysis of Multiple Phenolic Compounds in Amaranth (A. cruentus and A. tricolor)
Source: Molecules. 2020 Dec 1;25(23):5674. doi: 10.3390/molecules25235674 (PMC7730080; doi:10.3390/molecules25235674)
Supplement: Supplementary file 1 [file molecules-25-05674-s001.pdf]

molecules

Communication

Establishment of a UPLC-PDA/ESI-Q-TOF/MS-Based Approach for the Simultaneous Analysis of Multiple Phenolic Compounds in Amaranth (*A. cruentus* and *A. tricolor*)

Won Tea Jeong, Jun-Hyoung Bang, Seahee Han, Tae Kyung Hyun, Hyunwoo Cho, Heung Bin Lim * and Jong-Wook Chung *

Department of Industrial Plant Science and Technology, Chungbuk National University, Chungbuk, Cheongju 28644, Korea; shewaspretty@chungbuk.ac.kr (W.T.J.); peerage8794@gmail.com (J.-H.B.); saehee@gmail.com (S.H.); taekyung7708@chungbuk.ac.kr (T.K.H.); hwcho@chungbuk.ac.kr (H.C.)

* Correspondence: heungbin@chungbuk.ac.kr (H.B.L.); jakdukong@gmail.com (J.-W.C.)

Table S1. Sample information used in this study and concentration of phenolic compounds by UPLC-PDA.

IT Number	Species	Origin	Code	Parts	1	2	3	4	5	6	7	8	9	10	11	12	13	14	15	16	17
IT251901	*Amaranthus cruentus*	Ghana	ACL1	Leaf	0.00	0.56	2.74	1.25	2.26	1.56	0.29	0.00	0.65	2.94	219.68	42.38	0.75	6.07	1.70	0.16	0.53
			ACS1	Seed	0.00	3.58	0.00	0.00	0.82	0.58	0.00	0.00	0.00	0.00	0.00	0.00	0.00	0.00	0.00	0.00	0.81
IT251903	*Amaranthus cruentus*	Finland	ACL2	Leaf	1.18	0.59	2.01	1.99	0.94	0.97	0.25	0.13	0.74	0.76	203.68	12.52	0.65	5.96	1.71	0.27	0.77
			ACS2	Seed	0.00	2.54	0.00	0.00	0.00	0.00	0.00	0.00	0.00	0.00	3.39	0.00	0.00	0.00	0.00	0.00	17.41
IT199992	*Amaranthus tricolor*	Mexico	ATL1	Leaf	1.25	0.39	5.21	2.34	0.37	1.03	0.37	0.11	0.40	1.74	337.46	23.21	2.42	7.58	0.00	0.00	0.72
			ATS1	Seed	0.00	0.00	0.00	0.00	0.00	0.00	0.00	0.00	0.00	0.00	9.18	0.00	0.00	3.08	0.00	0.00	2.22
IT200005	*Amaranthus tricolor*	Mexico	ATL2	Leaf	1.17	0.65	7.26	2.38	2.29	1.48	0.46	0.10	0.40	1.43	150.21	18.40	1.30	5.30	2.00	0.21	0.41
			ATS2	Seed	0.00	0.00	0.00	0.00	0.00	0.00	0.00	0.00	0.00	0.21	9.65	0.00	0.00	0.00	0.00	0.00	0.00
IT200030	*Amaranthus tricolor*	Mexico	ATL3	Leaf	0.00	0.33	11.35	3.56	2.80	0.99	0.17	0.18	0.35	0.41	215.41	15.54	1.41	6.78	0.00	0.27	0.89
			ATS3	Seed	0.00	0.00	0.00	0.00	0.00	0.00	0.00	0.00	0.00	0.00	5.99	0.00	0.00	0.00	0.00	0.00	9.58
IT203379	*Amaranthus tricolor*	Cameroon	ATL4	Leaf	1.12	0.82	1.09	2.29	0.45	0.92	0.10	0.13	0.72	2.64	336.12	15.17	1.00	12.71	0.71	0.22	4.45
			ATS4	Seed	0.00	0.00	0.00	0.00	0.00	0.00	0.00	0.00	0.00	0.00	11.19	0.00	0.00	0.00	0.00	0.00	7.23

The number on the horizontal axis means the peak No of the compounds corresponding to figure 1.
